# Popular Science Monthly

**Published:** 1874-04

**Authors:** 


To those of our readers whose professional engagements permit the luxury of an occasional unbending from the strain of constant, uninterrupted practice, that they may stroll at will, and recreate themselves in the delightful fields of the collateral sciences, we heartily commend the *Popular Science Monthly;* the April No. of which lies before us. A hasty glancing over its pages has tempted us already to the neglect of pressing duties. Oh for a brief holiday, beyond the habitations of man, to drink in the balmy air of spring and hold sweet commune with nature, in the enlightened study of nature's laws!

The topics, in the present number, which attract our attention are: the Age of Ice, (illustrated); the Pathology of the Passions; Vivisection, by Prof. Foster; What the Chemistry of the Rocks Teaches; Evolution, and the Origin of Life. Subscription, $5 per year.

